# Pharmacokinetics, microscale distribution, and dosimetry of alpha-emitter-labeled anti-PD-L1 antibodies in an immune competent transgenic breast cancer model

**DOI:** 10.1186/s13550-017-0303-2

**Published:** 2017-07-18

**Authors:** Jessie R. Nedrow, Anders Josefsson, Sunju Park, Tom Bäck, Robert F. Hobbs, Cory Brayton, Frank Bruchertseifer, Alfred Morgenstern, George Sgouros

**Affiliations:** 1 Russell H. Morgan Department of Radiology and Radiological Science, Johns Hopkins University School of Medicine, CRBII 4M.61, 1550 Orleans Street, Baltimore, MD 21231 USA; 2 The Sahlgrenska Academy, University of Gothenburg, Gothenburg, Sweden; 3 Department of Radiation Oncology and Molecular Radiation Sciences, Johns Hopkins University School of Medicine, Baltimore, MD USA; 4 Department of Molecular and Comparative Pathobiology, Johns Hopkins University School of Medicine, Baltimore, MD USA; 5 European Commission Joint Research Centre, Directorate for Nuclear Safety and Security, Karlsruhe, Germany

**Keywords:** Pharmacokinetics, Dosimetry, Anti-PD-L1 antibodies, Immune checkpoint inhibition, Alpha-particle emitting radioimmunotherapy

## Abstract

**Background:**

Studies combining immune checkpoint inhibitors with external beam radiation have shown a therapeutic advantage over each modality alone. The purpose of these works is to evaluate the potential of targeted delivery of high LET radiation to the tumor microenvironment via an immune checkpoint inhibitor.

**Methods:**

The impact of protein concentration on the distribution of ^111^In-DTPA-anti-PD-L1-BC, an ^111^In-antibody conjugate targeted to PD-L1, was evaluated in an immunocompetent mouse model of breast cancer. ^225^Ac-DOTA-anti-PD-L1-BC was evaluated by both macroscale (ex vivo biodistribution) and microscale (alpha-camera images at a protein concentration determined by the ^111^In data.

**Results:**

The evaluation of ^111^In-DTPA-anti-PD-L1-BC at 1, 3, and 10 mg/kg highlighted the impact of protein concentration on the distribution of the labeled antibody, particularly in the blood, spleen, thymus, and tumor. Alpha-camera images for the microscale distribution of ^225^Ac-DOTA-anti-PD-L1-BC showed a uniform distribution in the liver while highly non-uniform distributions were obtained in the thymus, spleen, kidney, and tumor. At an antibody dose of 3 mg/kg, the liver was dose-limiting with an absorbed dose of 738 mGy/kBq; based upon blood activity concentration measurements, the marrow absorbed dose was 29 mGy/kBq.

**Conclusions:**

These studies demonstrate that ^225^Ac-DOTA-anti-PD-L1-BC is capable of delivering high LET radiation to PD-L1 tumors. The use of a surrogate SPECT agent, ^111^In-DTPA-anti-PD-L1-BC, is beneficial in optimizing the dose delivered to the tumor sites. Furthermore, an accounting of the microscale distribution of the antibody in preclinical studies was essential to the proper interpretation of organ absorbed doses and their likely relation to biologic effect.

## Background

The recent successes of novel immunomodulatory agents have brought immunotherapy up to the forefront as a possible treatment against various types of cancers [1–4]. This novel treatment approach is based on the observation that tumors avoid immune system recognition by co-opting immune checkpoints intended to prevent autoimmunity. One of the immune checkpoint mediators is the programmed cell death protein 1 (PD-1) receptor. PD-1 interacts with programmed cell death ligand 1 and 2 (PD-L1; PD-L2), with PD-L1 becoming a more commonly used target for immune checkpoint therapy [2, 5, 6]. PD-L1 is expressed on a variety of tumor cells, tumor-associated macrophages (TAM), and other cells in the tumor microenvironment [7, 8]. PD-1 expression on immune effector cells and PD-L1 expression on tumors and TAMs enables a receptor ligand interaction that leads to immune effector cell anergy. Antibodies (Ab) against PD-1 or PD-L1 block this interaction and activate anti-tumor immunity. Clinical trials of anti-PD-1 and anti-PD-L1 Ab have yielded promising results in patient populations that have exhausted all other conventional therapeutic options [2, 3].

A number of investigators have demonstrated the potential advantages of combined immune checkpoint inhibition therapy and external beam radiotherapy [9–11]. Radiation-induced inflammation enhances anti-tumor immunity by the release of intracellular proteins following radiation-induced cell death. The resulting increase in cytokine signaling enhances the expression of immune checkpoint targets, including PD-L1, thereby making the tumor more susceptible to checkpoint inhibition therapy. However, radiation dose delivery by external beam cannot be targeted at the cellular level. Cell-specific targeting is possible using targeted radiotherapy. With the exception of efforts to target tumor vasculature [12–14], the focus of such a targeted radiation delivery paradigm is to identify tumor-specific or tumor-associated antigens and use radiolabeled antibodies to specifically target tumor cells [15–19]. The anti-PD-L1 Ab conjugated to a therapeutic radionuclide would therefore target both a PD-L1-expressing tumor and PD-L1-positive cells in its microenvironment. Targeting PD-L1-positive cells in the tumor would reduce immune suppressive activity while also potentially enhancing immunity by the inflammatory and adjuvant release mechanisms outlined for external beam radiotherapy. Alpha-particle emitters are ideal in such a cell-specific targeting strategy because of their short, 50–100 μm range, and high potency. Furthermore, anti-PD-L1-targeted alpha-particle emitters would also be effective at eradicating metastatic cancer. Successful implementation of such a targeted radiotherapy/immunostimulatory strategy requires a detailed understanding of the microscale distribution of the PD-L1-targeted alpha-particle emitter labeled agent. In addition, the optimization of controllable variables such as the protein dose administered.

We previously reported on the feasibility of modifying an anti-PD-L1 antibody to deliver an imaging radionuclide in an immune-intact mouse model [20]. This work extends these studies to examine these additional variables towards optimal implementation of a combined targeted alpha-emitter/immune checkpoint blockade therapeutic strategy.

## Methods

### Reagents

All chemicals were purchased from Sigma-Aldrich Chemical Co. (St. Louis, MO, USA) or Thermo Fisher Scientific (Pittsburgh, PA, USA), unless otherwise specified. Aqueous solutions were prepared using ultrapure water (resistivity, 18 MΩ cm). p-SCN-Bn-DTPA and p-SCN-Bn-DOTA were purchased from Macrocyclics, Inc. (Dallas, TX, USA). [^111^In]InCl_3_ was purchased from MDS Nordion (Vancouver, BC, Canada). ^225^Ac was produced from a ^229^Th source as described in [21, 22]. The *InVivo*Plus anti-mouse PD-L1 Ab (anti-PD-L1-BC) was purchased from BioXCell (West Lebanon, NH, USA). Blood chemistry was determined using a Spotchem EZ Vet from Scil Animal Care Company (Gurnee, IL, USA).

### Radiolabeling

The anti-PD-L1-BC Ab was conjugated to *N*-[2-amino-3-(*p*-isothiocyanatophenyl)propyl]-*trans*-cyclohexane-1,2-diamine-*N*,*N′*,*N′*,*N″*,*N″*-pentaacetic acid (SCN-CHX-A*″*-DTPA) using previously described standard methods yielding DTPA-anti-PD-L1 [23, 24]. Briefly, [^111^In]InCl_3_ (37-74 MBq) was added to an acid washed 1.5 mL eppendorf tube containing 0.25 mL of 0.2 M HCl and 0.03 mL of 3 M NH_4_OAc, pH = 7. After a minute, 0.2 mg of DTPA-anti-PD-L1-BC Ab was added to the mixture. The mixture was allowed to set at room temperature for 45–60 min and then transferred to an Amicon Ultra 10K centrifugal filter device. Phosphate-buffered saline (PBS) was added and the device was centrifuged for 15 min at 3000 rpm to remove free [^111^In]InCl_3_ (1×). Radiochemical purity of >98% was determined by radio TLC, and the protein concentration was determined by Nanodrop (Wilmington, DE, USA).

The ^225^Ac-labeled anti-PD-L1-BC Ab conjugate was prepared using the high-efficiency one-step method described in PCT/US2010/042885. For the one-step method, p-SCN-Bn-DOTA was conjugated to the anti-PD-L1-BC Ab overnight and the resulting conjugate, DOTA-anti-PD-L1-BC, was purified prior to radiolabeling. DOTA-anti-PD-L1-BC (0.5 mg) was then radiolabeled with ^225^Ac (23 MBq) in 100 μL of 3 M sodium acetate at 37 °C for 90 min. The resulting radiolabeling conjugate ^225^Ac-DOTA-anti-PD-L1-BC was purified using a 10 DG desalting column (Bio-Rad, Hercules, CA, USA) prepped with PBS containing 1% bovine serum albumin (BSA) followed by a PBS wash. The radiolabeled conjugate was eluted with PBS and collected in 0.5 mL fractions. TLC determined radiochemical purity, and the protein concentration was determined by Nanodrop (Wilmington, DE, USA).

### In vivo studies

#### Cell lines

The NT2.5 cell line was established from spontaneous mammary tumors in female *neu*-N mice [25, 26]. Dr. Elizabeth Jaffee, Johns Hopkins University, kindly provided the NT2.5 cell line in 2014. All cells were confirmed to be Mycoplasma negative (Hoechst stain and PCR; tested in 2014). The NT2.5 cell lines obtained was not authenticated by the Sgouros lab. NT2.5 cells were grown in RPMI1640 media with 20% fetal bovine serum (FBS) + 1.2% HEPES + 1% l-glutamine + 1% non-essential amino acids (NEAA) + 1% sodium pyruvate + 0.2% insulin + 0.02% gentamycin. Cells were cultured for a maximum of 4 weeks before thawing fresh, early passage cells.

#### Animals

Animal studies were performed using FVB/N-Tg(MMTVneu)202Mul/J (*neu*-N) female mice, 4 to 7 weeks old, purchased from The Jackson Laboratory (Bar Harbor, ME, USA). All animal studies were approved by the Animal Care and Use Committee of the Johns Hopkins University, School of Medicine.

#### Biodistribution of ^111^In- and ^225^Ac-labeled anti-PD-L1-BC antibodies in tumor-bearing neu-N mice

Biodistribution experiments were conducted as previously described with minor modifications [18, 27, 28]. Briefly, *neu*-N mice (*n* = 3–5/time point) were injected subcutaneously (s.c.) with NT2.5 cells (1 × 10^6^) in the right flank. Following a growth period of 4 weeks, the tumor-bearing mice were injected intravenously (i.v.) with ^111^In-DTPA-anti-PD-L1-BC (370 kBq) at the following Ab protein concentrations: 1, 3, and 10 mg/kg as well as with ^225^Ac-DOTA-anti-PD-L1-BC (15 kBq) at 3 mg/kg. At 1, 6, 24, 72, and 144 h post-injection (p.i.), the mice were sacrificed. The blood, heart, lungs, liver, spleen, kidneys, stomach (with content), intestine (with content), bone, thymus, muscle, tumor, and brown adipose tissue (BAT) were harvested, weighed, and measured in a gamma well counter (PerkinElmer 2470 WIZARD2® Automatic Gamma Counter, MA, USA) using the 400 to 480 keV energy window for ^213^Bi (440.6 keV and yield 26.1%). The percent-injected activity per gram (%IA/g) was calculated by comparison to a weighed, diluted standard.

#### Alpha-camera imaging, histology, and immunohistochemistry

The alpha-camera is an ex vivo digital autoradiography imaging technique dedicated to detecting emitted alpha-particles [29]. The alpha-camera was used to image the distribution and relative activity concentrations of ^225^Ac-DOTA-anti-PD-L1-BC within the normal tissues and tumors. *neu*-N mice (*n* = 1/time point) were injected s.c. with NT2.5 cells (1 × 10^6^) in the right flank. Following a growth period of 4 weeks, the tumor-bearing mice were injected i.v. with ^225^Ac-DOTA-anti-PD-L1-BC (67 kBq). The mice were sacrificed at 1, 6, 24, and 72 h, and the organs (spleen, kidney, liver, thymus, and tumor) were immediately removed, embedded in optimal cutting temperature (OCT) compound and frozen (−78.5 °C). Consecutive, 8-μm-thick cryostat sections of each tissue were obtained for alpha-camera imaging, hematoxylin and eosin (H&E) staining, and immunohistochemistry. Immunohistochemistry was performed for CD8 (anti-mouse CD8a, 4SM15, eBioscience). Exposure times for alpha-camera imaging were in the range of 20 to 24 h. Alpha-camera images were analyzed using the software, ImageJ 1.49 g (National Institutes of Health, Bethesda, MD, USA).

#### Normal tissue and tumor dosimetry

Normal tissue and tumor mean absorbed doses were calculated using the ^225^Ac-DOTA-anti-PD-L1-BC biodistribution data. The radioactivity concentration vs time curves were fitted using the software SAAM II (The Epsilon group, Charlottesville, VA, USA) and integrated from zero to infinity to calculate the time-integrated activity per unit mass. If the data could not be fitted, the integral was obtained as the sum of a numerical integration over the measured time period and an analytically integrated exponential function based on an exponential extrapolation beyond the last measure time point that used the log-linear slope of the last two time points as the exponential clearance rate. The mean absorbed dose $\overline{D}$ to the normal tissues and tumors was calculated using the following expression:

1 $$ \overline{D}=\tilde{A}\cdot \frac{\varDelta \cdot \phi}{m} $$

where *Ã* is the time-integrated activity, $m$ is the weight of the normal tissue or tumor, $\mathrm{\triangle}\textcolor[rgb]{1,0,0}{\backslash}\textcolor[rgb]{1,0,0}{\mathrm{increment}}$ is the mean energy per nuclear transition, and $\varnothing$ is the absorbed fraction [30]. For ^225^Ac, $\mathrm{\triangle}\textcolor[rgb]{1,0,0}{\backslash}\textcolor[rgb]{1,0,0}{\mathrm{increment}}$ = 4.40E−12 J/(Bq s), which includes the mean energy per nuclear transition from the descendants ^221^Fr, ^217^At, ^213^Bi, and ^213^Po, and for ^213^Bi, $\mathrm{\triangle}\textcolor[rgb]{1,0,0}{\backslash}\textcolor[rgb]{1,0,0}{\mathrm{increment}}$ = 1.33E−12 J/(Bq s) [31]. Only the emitted alpha-particles were considered in the dosimetric calculations, and all energy was assumed to be deposited locally ($\varnothing =1$). Decay of ^225^Ac leads to the release of free ^213^Bi, which concentrates in the kidneys [19, 32]. Accordingly, the dose contribution to kidneys from free ^213^Bi was added to the ^225^Ac-absorbed dose. The radioactivity in kidneys was measured using a gamma well counter. Counts detected over a 1-min interval were recorded for 4.5 h. The measurements were fitted with a double exponential function from which the activity per unit mass (Bq/g) of free ^213^Bi (obtained as the time-zero intercept) and ^225^Ac-anti-PD-L1-BC within the kidneys at the time of sacrifice (obtained once the decay rate corresponds to the 10-day half-life of ^225^Ac) could be determined.

To assess the impact of protein amount administered on the optimal alpha-particle emitter choice, the time-integrated activity (TIA) for the tumor, blood, spleen, liver, kidneys, and thymus were calculated using the ^111^In-DTPA-anti-PD-L1-BC 1, 3, and 10 mg/kg biodistribution data. These data were used to calculate the TIA for three different radionuclides that are candidates for alpha-particle emitter therapy: ^225^Ac, ^212^Pb, and ^211^At with half-lives of 10 days, 10.6 h, and 7.2 h, respectively.

#### Max tolerated dose (MTD)

Healthy non-tumor-bearing *neu*-N mice (*n* = 5 mice/group) were injected i.v. with ^225^Ac-DOTA-anti-PD-L1-BC (3 mg/kg) at the following doses: 15, 22, 30, 37, and 44 kBq. The dose range was based on previously performed MTD studies with ^111^In-DTPA-7.16.4 as well as organ and blood clearance rates of ^111^In-DTPA-anti-PD-L1-BC in comparison to ^111^In-DTPA-7.16.4 (data not shown) [19]. The mice were monitored for signs of short-term toxicity for 100 days. For each mouse, the study was ended and the mouse sacrificed when one of the following conditions was met: (1) greater than 20% weight loss, (2) evidence of pain or distress, and (3) 100 day endpoint. At time of sacrifice, necropsies were performed: the spleen, kidney, liver, and thymus were removed, weighed, and sent for histopathology. Histopathological analysis was evaluated on sections of these organs stained with H&E and Periodic acid-Schiff staining (PAS) (kidney and liver); Johns Hopkins Medical Laboratories embedded, sectioned, and stained reported tissues. The following blood chemistries were performed for the lowest and highest dose groups: blood urea nitrogen (BUN), alkaline phosphatase (ALP), total protein (T-Pro), alanine aminotransferase (ALT), creatinine (Cre), and calcium (Ca).

### Statistical analysis

Statistical analysis was performed using the software, GraphPad (La Jolla, CA USA). Groups were compared using multiple *t* test and two-way and one-way ANOVAs. *p* values were considered significant if *p* ≤ 0.05.

## Results

### Radiochemistry

The ^111^In-DTPA-anti-PD-L1-BC conjugate was radiolabeled in 45–60 min at room temperature at a specific activity of 11.8 MBq/nmol with >95% radiochemical purity following purification. The ^225^Ac-DOTA-anti-PD-L1-BC conjugate was radiolabeled in 90 min at 37 °C at a specific activity of 4.0 ± 0.7 MBq/nmol with >95% radiochemical purity following purification.

### In vivo studies

#### Biodistribution of ^111^In-DTPA-anti-PD-L1-BC and ^225^Ac-DOTA-anti-PD-L1-BC antibodies in tumor-bearing neu-N mice

**Fig. 1:**
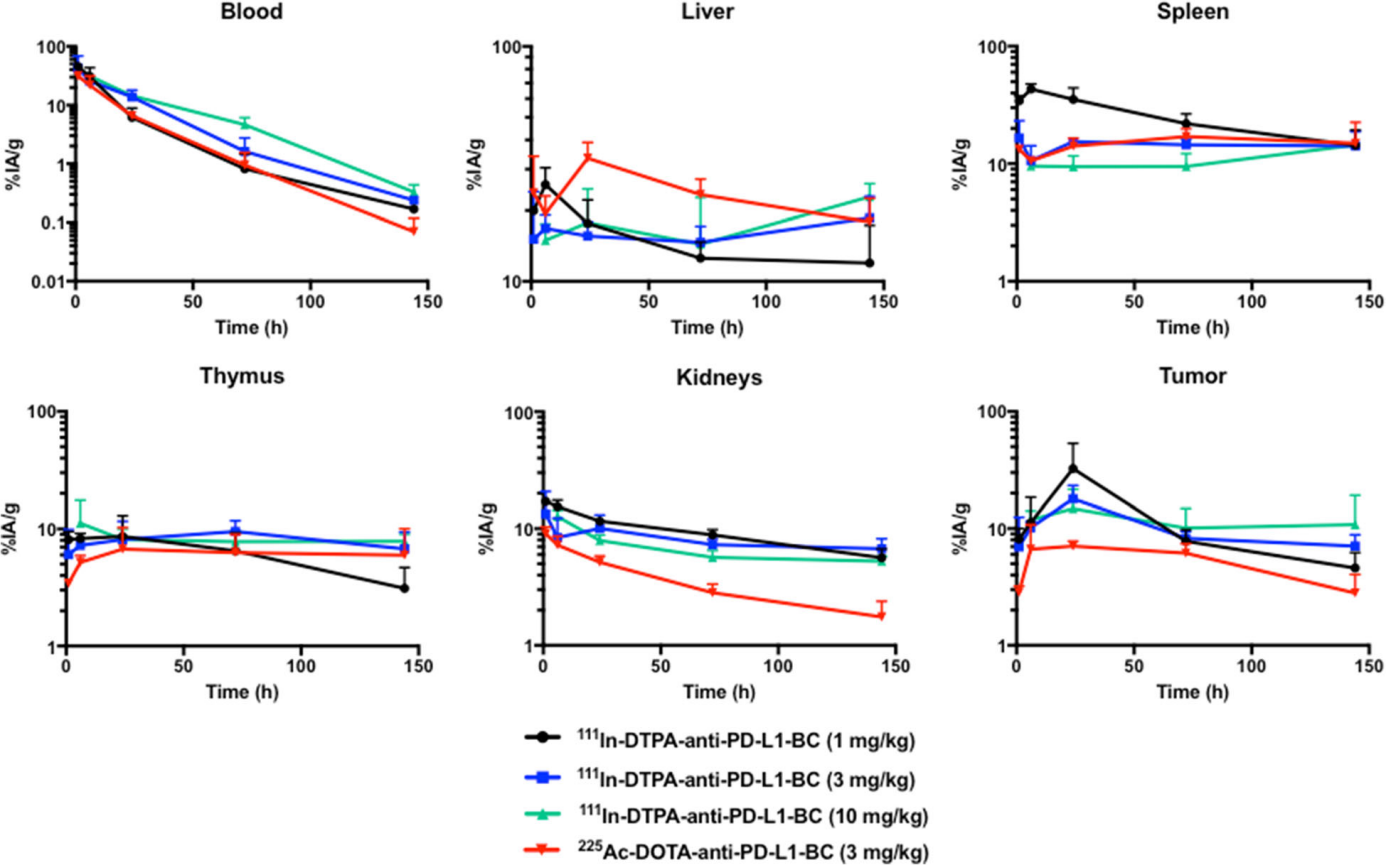
Clearance profiles of select organs: Blood, liver, spleen, thymus, kidneys, and tumor (note that the *y*-axis is presented on a logarithmic scale)

**Fig. 2:**
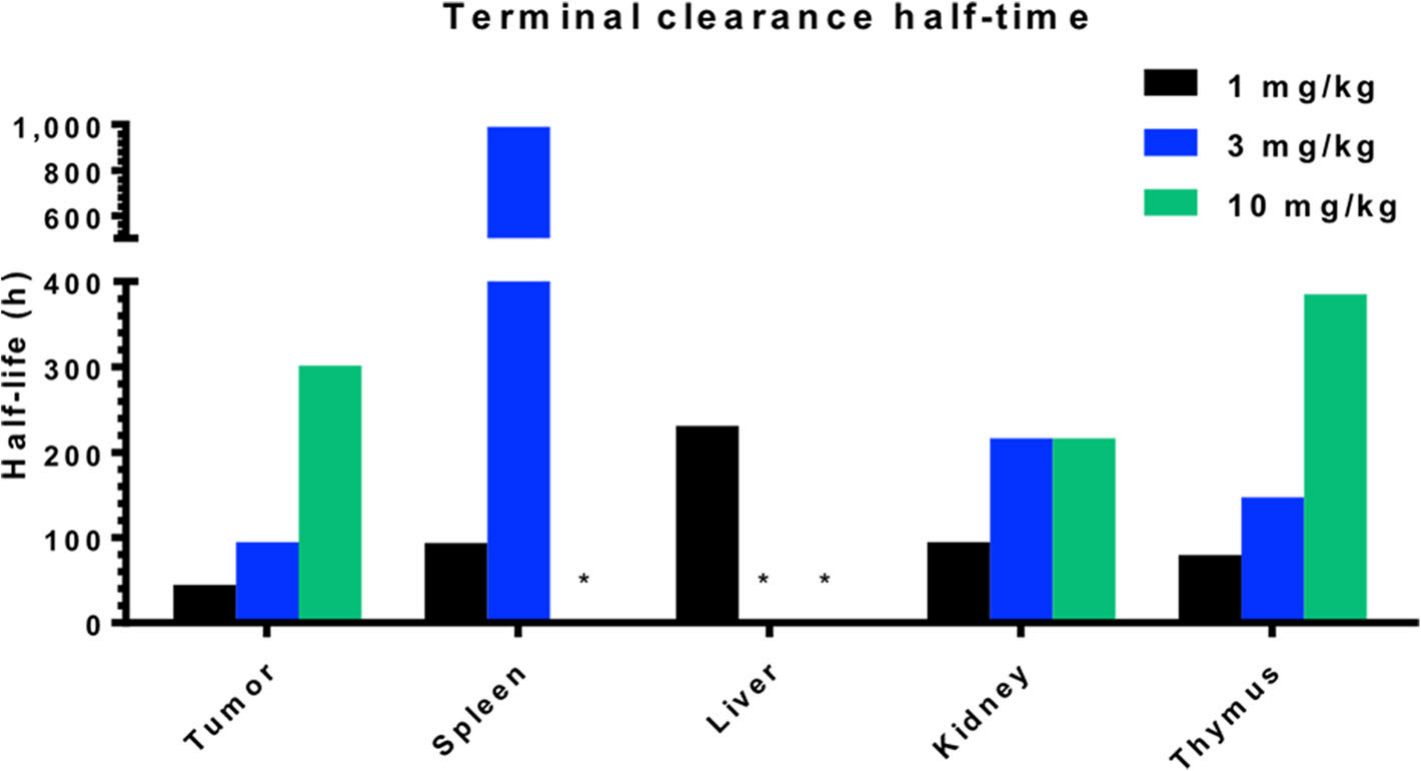
Terminal clearance half-time, calculated from last two measured time points

**Fig. 3:**
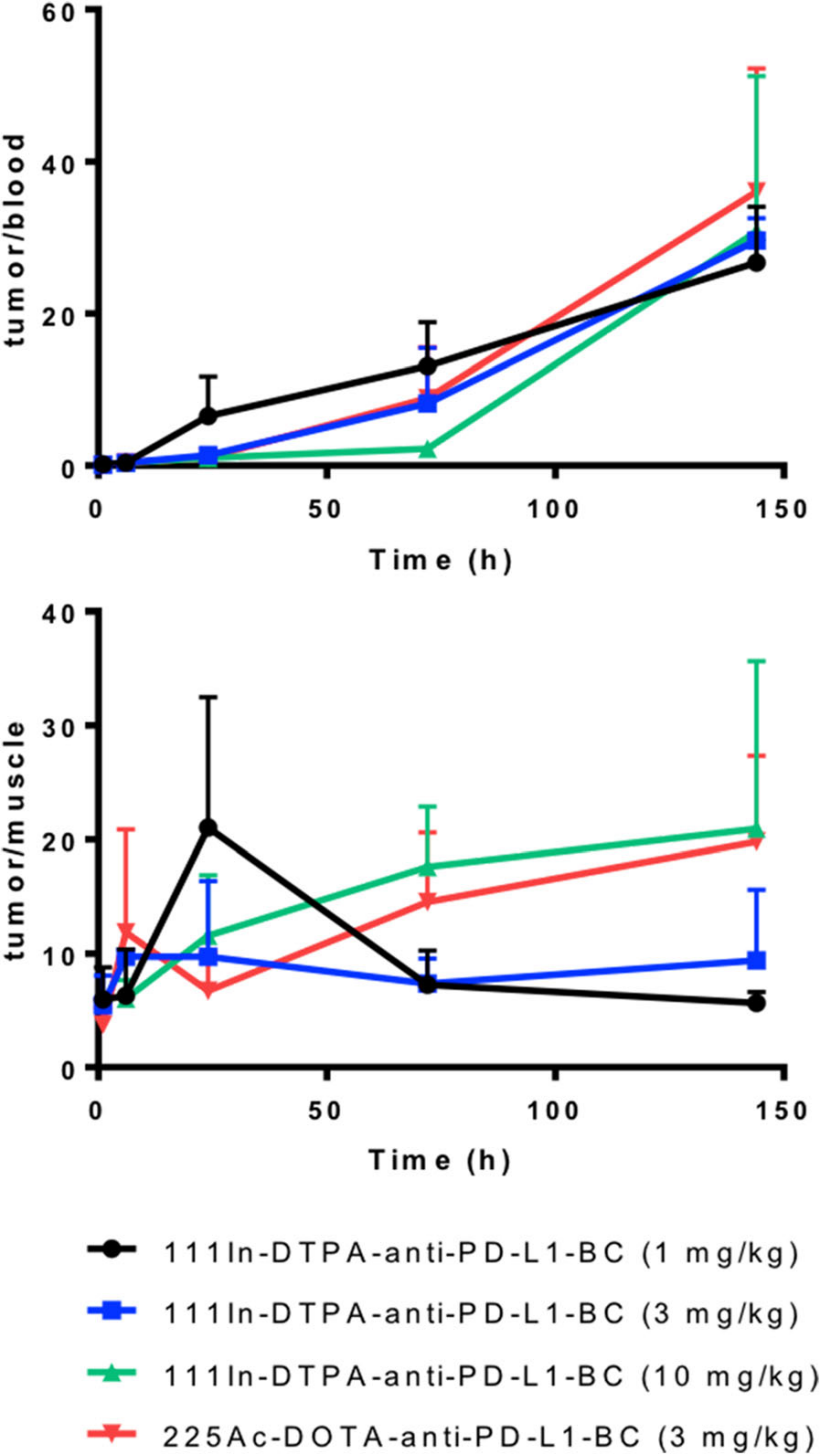
Tumor to blood and tumor to muscle activity concentration ratios for individual agents

**Fig. 4:**
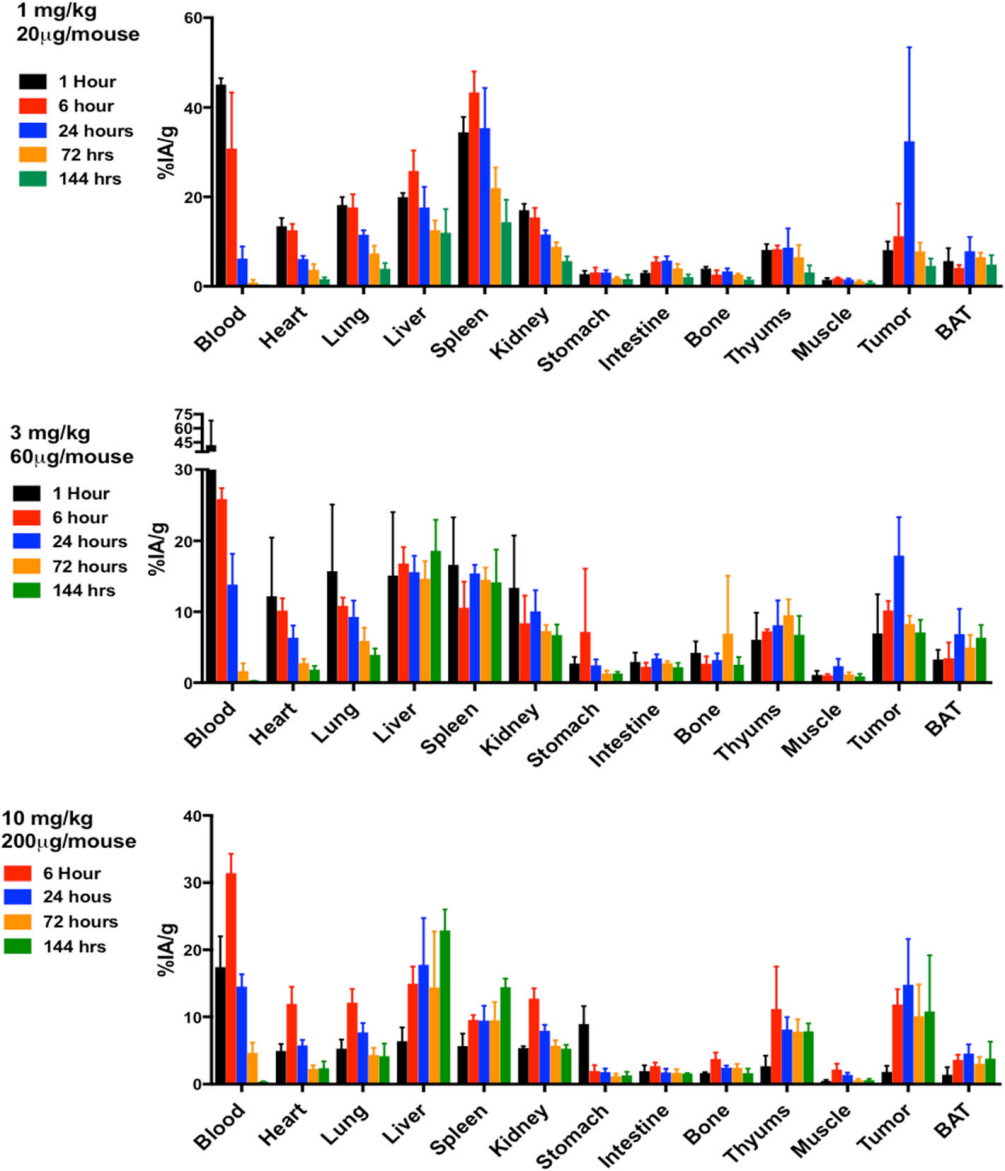
Biodistribution of ^111^In-DTPA-anti-PD-L1-BC (370 kBq) at 1, 6, 24, 72, and 144 h in *neu*-N mice bearing NT2.5 tumors. ^111^In-DTPA-anti-PD-L1-BC was administered at the following doses: 1 mg/kg (20 μg/mouse), 3 mg/kg dose (60 μg/mouse), and 10 mg/kg (200 μg/mouse)

**Fig. 5:**
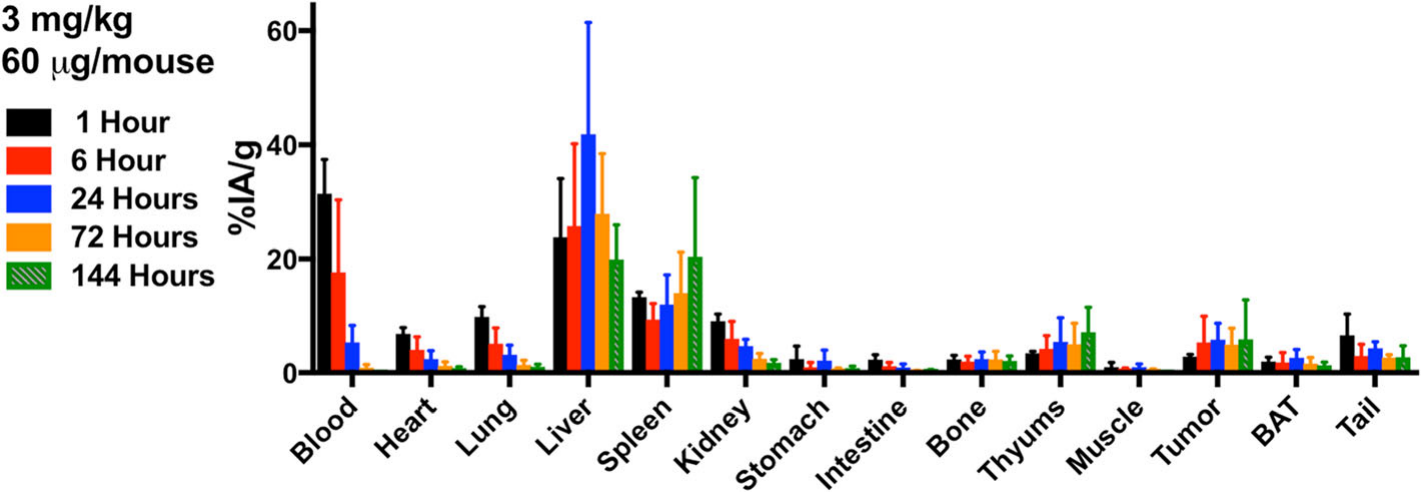
Biodistribution of ^225^Ac-DOTA-anti-PD-L1-BC (15 kBq) at 1, 6, 24, 72, and 144 h in *neu*-N mice bearing NT2.5 tumors. ^225^Ac-DOTA-anti-PD-L1-BC was administered at the 3 mg/kg dose (60 μg/mouse)

The impact of Ab protein dose on the pharmacokinetics of anti-PD-L1 Ab was examined using ^111^In-DTPA-anti-PD-L1-BC (approximately 370 kBq) at the following protein doses: 1 mg/kg (20 μg Ab), 3 mg/kg (60 μg Ab), and 10 mg/kg (200 μg Ab); the pharmacokinetics of ^225^Ac-DOTA-anti-PD-L1-BC (15 kBq on 3 mg/kg) were also examined (Fig. 1). Blood pharmacokinetic parameters for each agent and protein dose are summarized in Table 1. Bi-phasic blood clearance was observed for the 1 and 3 mg/kg ^111^In-anti-PD-L1 Ab doses. At both doses, clearance is dominated (93 and 95%, respectively) by a rapid (7- and 14-h half-life, respectively) phase. A lower fraction of the ^225^Ac-DOTA conjugate is cleared rapidly (63% with a 6-h half-life), and correspondingly, a larger component (37%) clears with a 19-h half-life. The difference in blood kinetics is consistent with the PK differences of the DOTA- compared to the DTPA-conjugate seen in the liver, kidneys, and tumor (Fig. 1). Increasing protein dose leads to prolonged sequestration of the anti-PD-L1 Ab in the tumor, spleen, liver, and thymus (Fig. 2). Antibody is still being concentrated in the spleen at the 10 mg/kg dose. In the liver, uptake continues throughout the 6-day measurement period at both the 3 and 10 mg/kg dose. The tumor to blood and tumor to muscle concentration ratios as a function of time are important in identifying optimal imaging time for a potential theranostic treatment approach; Fig. 3 shows these ratios as a function of time. As expected for an intact Ab, the tumor to blood ratio increases with time for all protein doses. The tumor to muscle ratio is somewhat dependent on protein dose; the 10 mg/kg DTPA conjugate increases with time. At 6 days after i.v. administration, both tumor to normal tissue ratios are at a maximum for the 3 mg/kg ^225^Ac-DOTA conjugate. Biodistribution data for all tissues measured are provided in Figs. 4 and 5.

**Table 1:** Summary of blood pharmacokinetics

Construct/protein dose (mg/kg)	Percent	Half-life (h)
^111^In-DTPA-anti-PD-L1-BC/1	93	7
	7	32
^111^In-DTPA-anti-PD-L1-BC/3	95	14
	5	46
^111^In-DTPA-anti-PD-L1-BC/10^a^	100	21
^225^Ac-DOTA-anti-PD-L1-BC/3	63	6
	37	19

^a^1-h measurement not available, blood curve was fit to a single-phase exponential function

#### Alpha-camera imaging, histology, and immunohistochemistry

The short 50 to 100 μm range of alpha-particles requires an understanding of the microscale distribution of alpha-particle emitter labeled Ab. Accordingly, the macroscopic biodistribution of anti-PD-L1 Ab was supplemented with alpha-camera images depicting the microscale distribution.

**Fig. 6:**
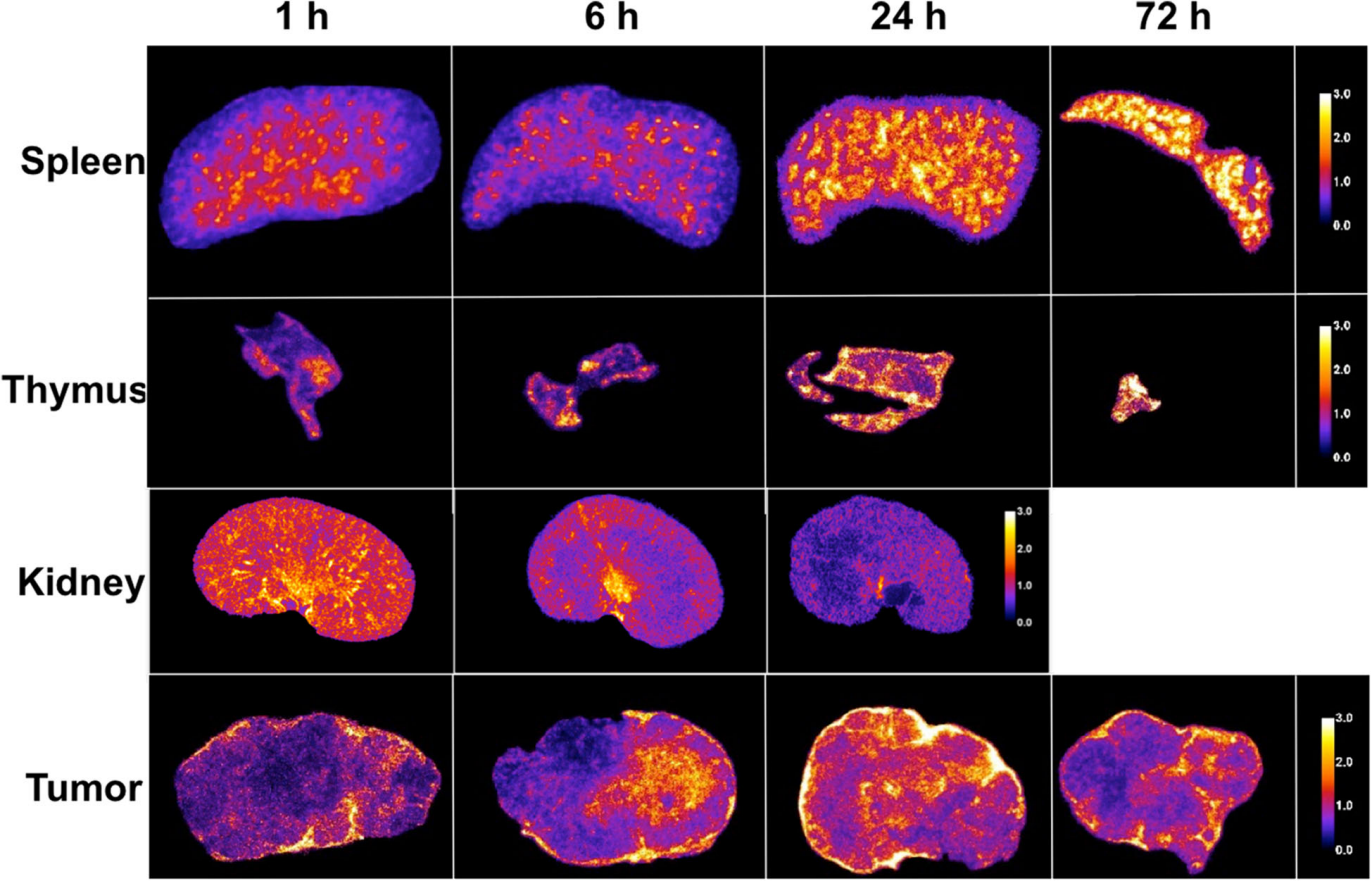
Alpha-camera images for the spleen, thymus, kidney, and tumor following i.v. administration of ^225^Ac-DOTA-anti-PD-L1-BC (67 kBq, 3 mg/kg). The scale is normalized for each tissue so that the average intensity across each row is set to 1. A 72-h kidney image and 144-h images for all other organs could not be obtained due to low counts

The microscale distribution of ^225^Ac-DOTA anti-PD-L1 Ab in several tissues and in tumors is shown over time in Fig. 6. Even though a single 8-μm slice is shown at each time point and for each tissue, the intensity at each time point corresponds qualitatively to the time vs activity concentration curves shown on Fig. 1 for the ^225^Ac-DOTA Ab. For example, the spleen appears more intense at 1 than at 6 h and progressively increases in intensity thereafter. Likewise, the thymus is least intense at 1 h and then reaches maximum intensity by 24 h. Only a small piece of the thymus could be imaged at 72 h p.i.; the average intensity of this piece, however, is approximately the same as at 24 h.

**Fig. 7:**
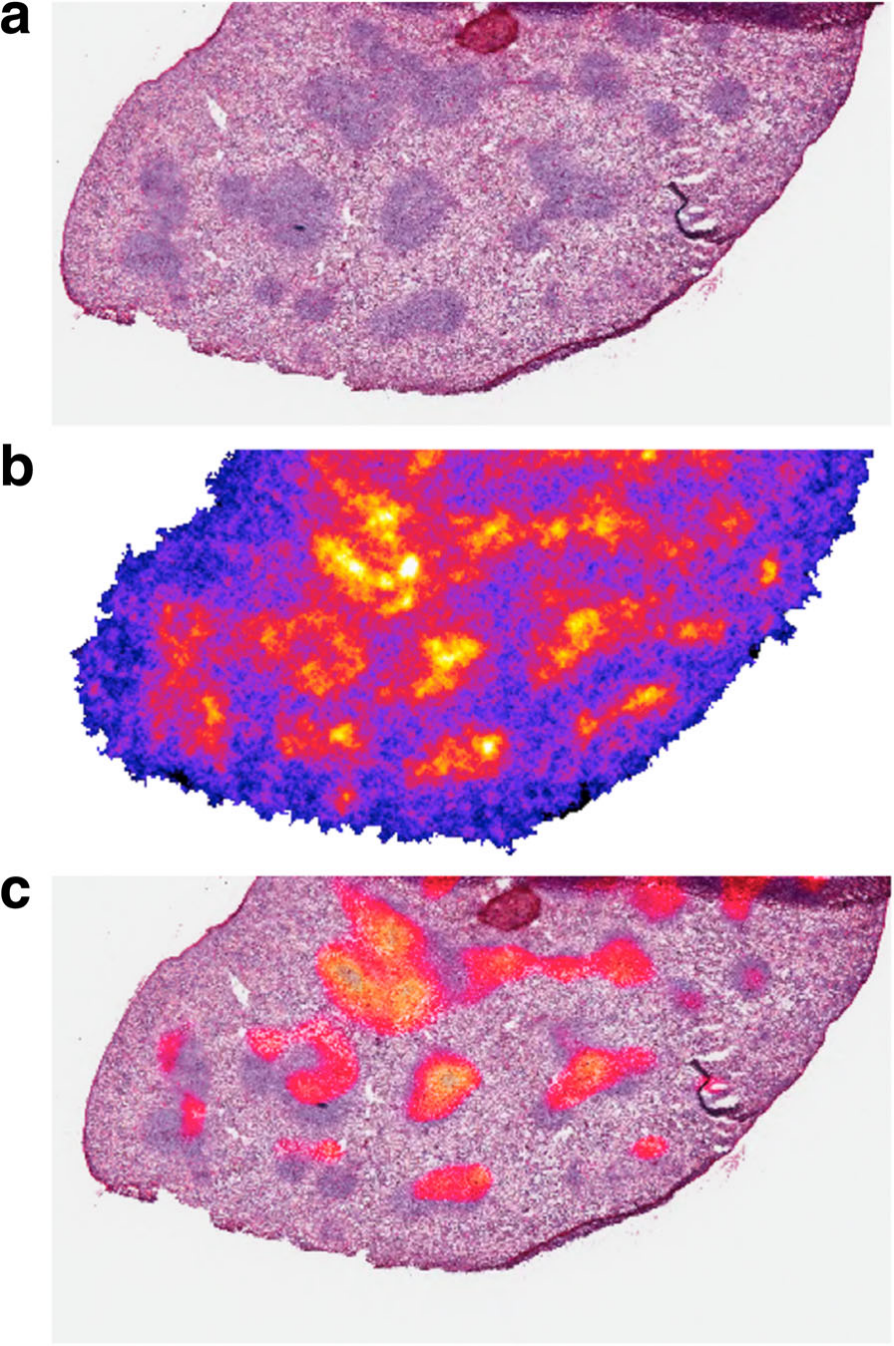
Sub-region of the spleen showing **a** the lymphocyte-rich, *white* pulp region (purple staining on H&E). **b** The corresponding alpha-camera and **c** a merged image showing segmented high-intensity regions overlayed on the H&E-stained slice. Segmented regions do not exactly match the purple white pulp region due to differences in tissue processing, and the comparison is being made across two different 8-μm-thick slices

**Fig. 8:**
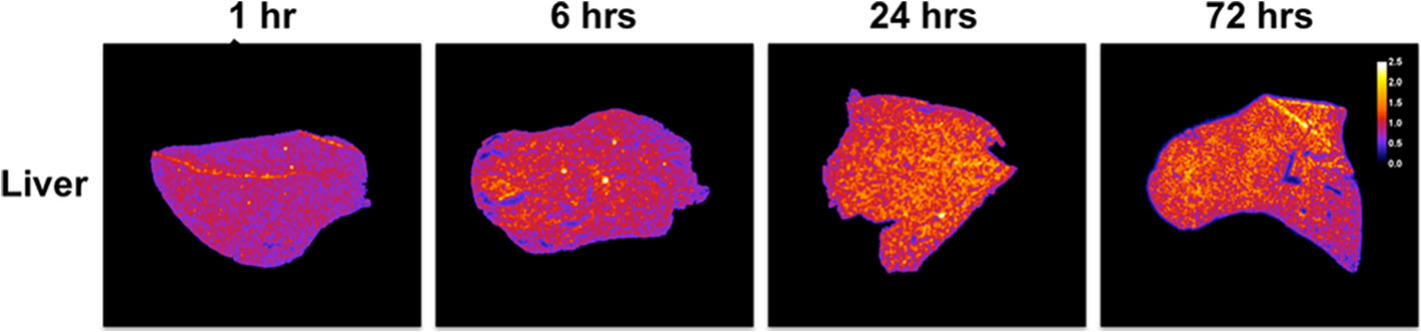
Alpha-camera images for the liver following IV administration of ^225^Ac-DOTA-anti-PD-L1-BC (67 kBq, 3 mg/kg). The scale is normalized so that the average intensity over time is set to 1

The microscale distribution in the spleen and thymus is consistent with targeting lymphocyte-rich regions. In the spleen, the anti-PD-L1 Ab distribution at 24 h corresponds to white-pulp regions (Fig. 7). The white pulp regions are predominately comprised of lymphocytes, antigen-presenting cells, and macrophages in addition to T and B cells [33]. PD-L1 is shown to be expressed by normal splenic cells as well as B cells, dendritic cells, macrophages, and NK cells [7, 8]. In the thymus, the highest intensity is in the cortex region of the thymus, which has a higher lymphocyte density than other regions. This is most clearly seen in the 24-h section with up to three times higher activity concentrations in the cortex, which is associated with CD4/CD8 double-negative thymocytes that have high expression of PD-L1 [8]. The distribution pattern in the kidneys for ^225^Ac-DOTA Ab is consistent with the 150 kD molecular weight of Ab which precludes them from glomerular filtration. After an initial grossly uniform distribution, the labeled antibody is primarily in the collecting ducts with some fraction still in the cortex at 6 h. By 24 h, the entire signal is in the collecting ducts. The distribution in the liver also followed the macroscopic kinetic data and was generally uniform (Fig. 8).

**Fig. 9:**
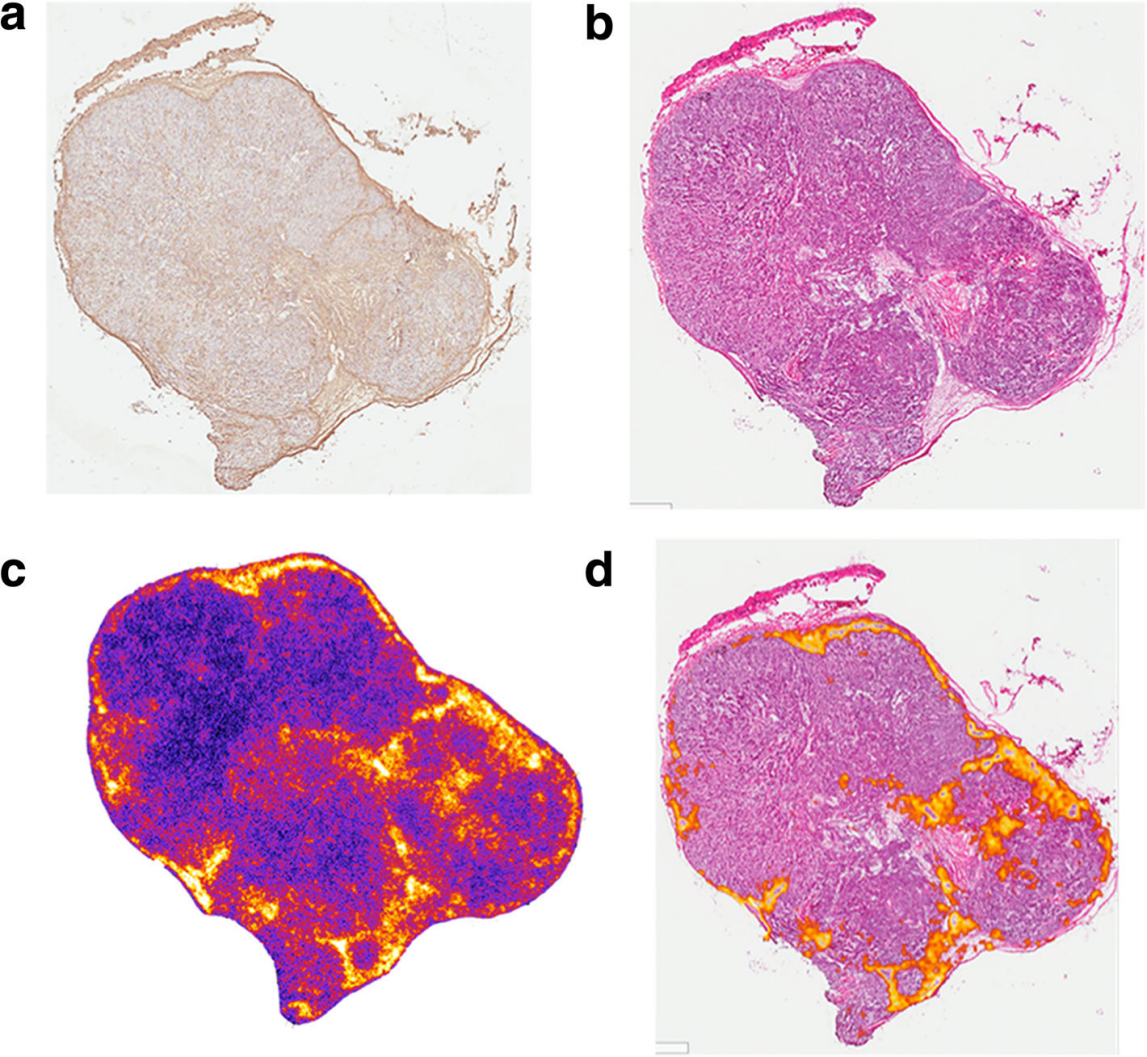
Four sections of a tumor collected 72 h after ^225^Ac-DOTA-anti-PD-L1-BC antibody administration. **a** CD8 immunohistochemistry and **b** H&E stain. **c** Alpha-camera and **d** segmented high-intensity alpha-camera regions overlayed on the H&E-stained section

Alpha-camera images of the tumor have intensity at each time point that is also consistent with the macroscopic measurements depicted on Fig. 1. The Ab distribution within each tumor is non-uniform at all times p.i.; peak intensity is seen at 24 h. At this time, Ab has penetrated throughout most of the tumor section and there is also very high intensity at the periphery. The 72-h tumor section has a lower overall intensity. Antibody is distributed in focal high-intensity regions corresponding primarily to stromal regions of the tumor. Although stromal regions also had generally high CD8 staining on immunohistochemistry, this was not clearly associated with cellular membranes and is likely to be non-specific accumulation. There was very modest evidence of immune infiltration of the tumor. The distribution of ^225^Ac-DOTA-anti-PD-L1 Ab in this tumor at 72 h after i.v. injection is most likely a combination of binding to accessible PD-L1-positive cells, in the stromal region, and the vascular architecture of the tumor (Fig. 9).

#### Normal tissue and tumor dosimetry

**Fig. 10:**
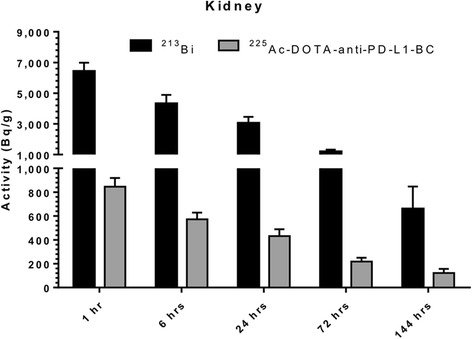
Activity per unit mass (Bq/g) in kidneys for free ^213^Bi (*black bar*) and ^225^Ac-DOTA-anti-PD-L1-BC (*gray bar*) at 1, 6, 24, 72, and 144 h p.i. from a 15-kBq ^225^Ac-DOTA-anti-PD-L1-BC (3 mg/kg, 60 μg) i.v. injection

The mean absorbed doses from ^225^Ac-DOTA-anti-PD-L1-BC for the normal tissues and tumor are shown in Table 2. The liver and spleen received the highest absorbed doses: 738 and 615 mGy/kBq, respectively; the kidneys received 138 mGy/kBq with 86.6 mGy/kBq (63%) due to free ^213^Bi (Fig. 10). The mean absorbed dose to the tumor was 141 mGy/kBq. Although the liver and spleen absorbed doses are higher than the tumor absorbed dose, the spleen is not a vital organ and the liver can tolerate 27 to 32 Gy of low LET radiation (twice daily fractions, 1.5 Gy/fraction) while 18–23 Gy external radiotherapy to the whole kidney volume gives 5% risk of kidney injury in 5 years [34, 35]. Hematopoietic toxicity is dose limiting for most radiolabeled immunoconjugate therapy. The blood absorbed dose may be used to estimate marrow absorbed dose for intact antibody. Based on rapid equilibration and volume of distribution assumptions, the red marrow absorbed dose is approximately 36% of the blood absorbed dose [36]. Applying this assumption, the red marrow absorbed dose per unit-administered activity is 29 mGy/kBq. The limit on the amount of activity that may be administered for therapy is determined by the liver. Assuming a liver MTD of 28 Gy, 38 kBq could be administered to deliver a tumor absorbed dose of 5.3 Gy. This activity would give a marrow absorbed dose of 1 Gy, which is below the 2 to 3 Gy limit for hematologic toxicity [37].

**Table 2:** Mean absorbed doses to normal organs and tumor for ^225^Ac-DOTA-anti-PD-L1-BC (15 kBq, 3 mg/kg)

Organ	Mean absorbed dose (mGy/kBq)
Blood	80.4
Heart	42.5
Lungs	53.0
Liver	738
Spleen	615
Kidneys^a^	138
Stomach	30.2
Intestine	42.8
Bone	69.7
Thymus	282
Muscle	11.8
BAT	57.5
Tumor	141

^a^Includes contribution from free ^213^Bi and ^225^Ac-DOTA-anti-PD-L1-BC

**Fig. 11:**
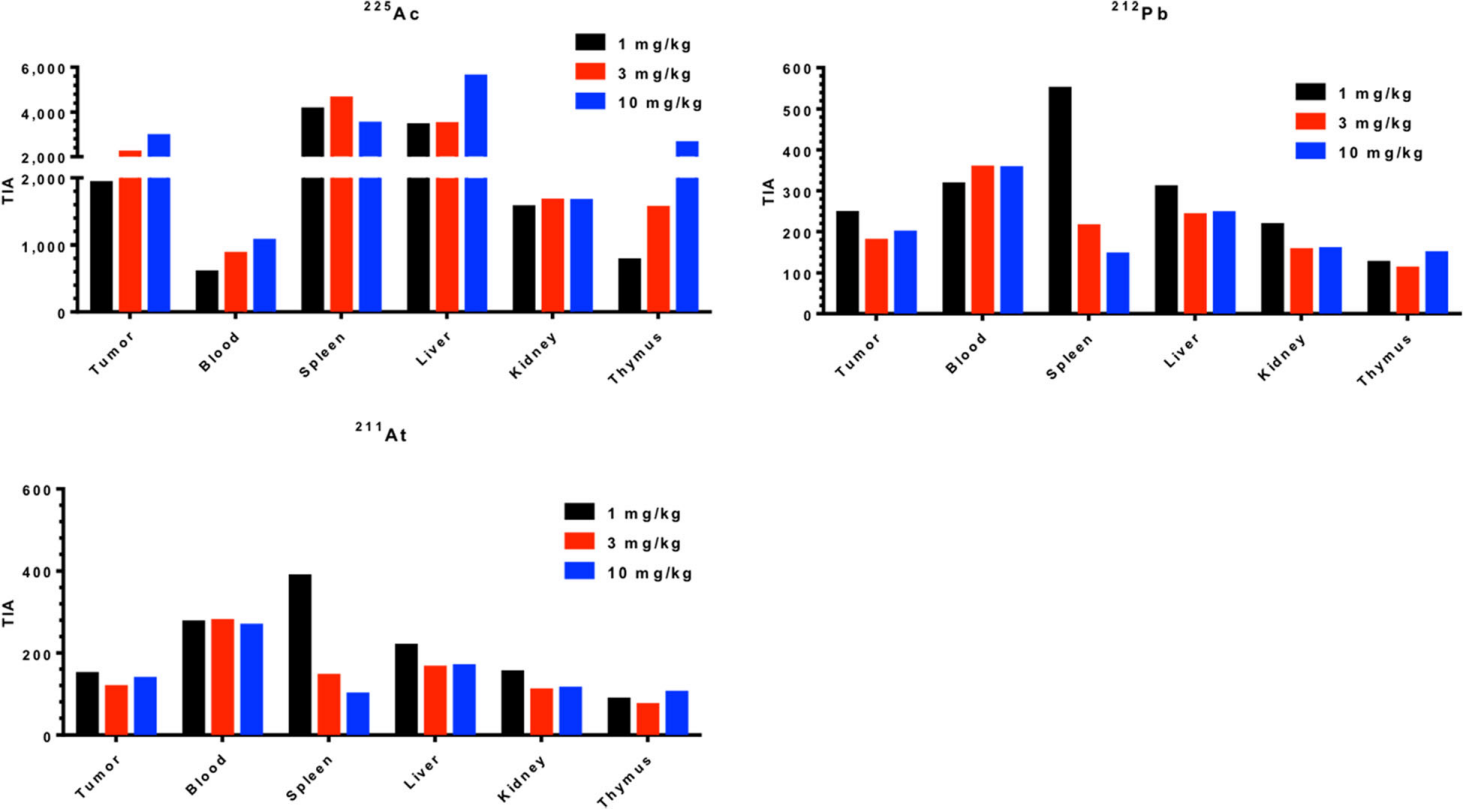
Time-integrated activity (TIA) of the tumor, blood, spleen, liver, kidney, and thymus for the radionuclides **a** ^225^Ac, **b** ^212^Pb, and **c** ^211^At-anti-PD-L1-BC labeled with the protein concentrations 1 mg/kg (*black bar*), 3 mg/kg (*red bar*), and 10 mg/kg (*blue bar*)

**Fig. 12:**
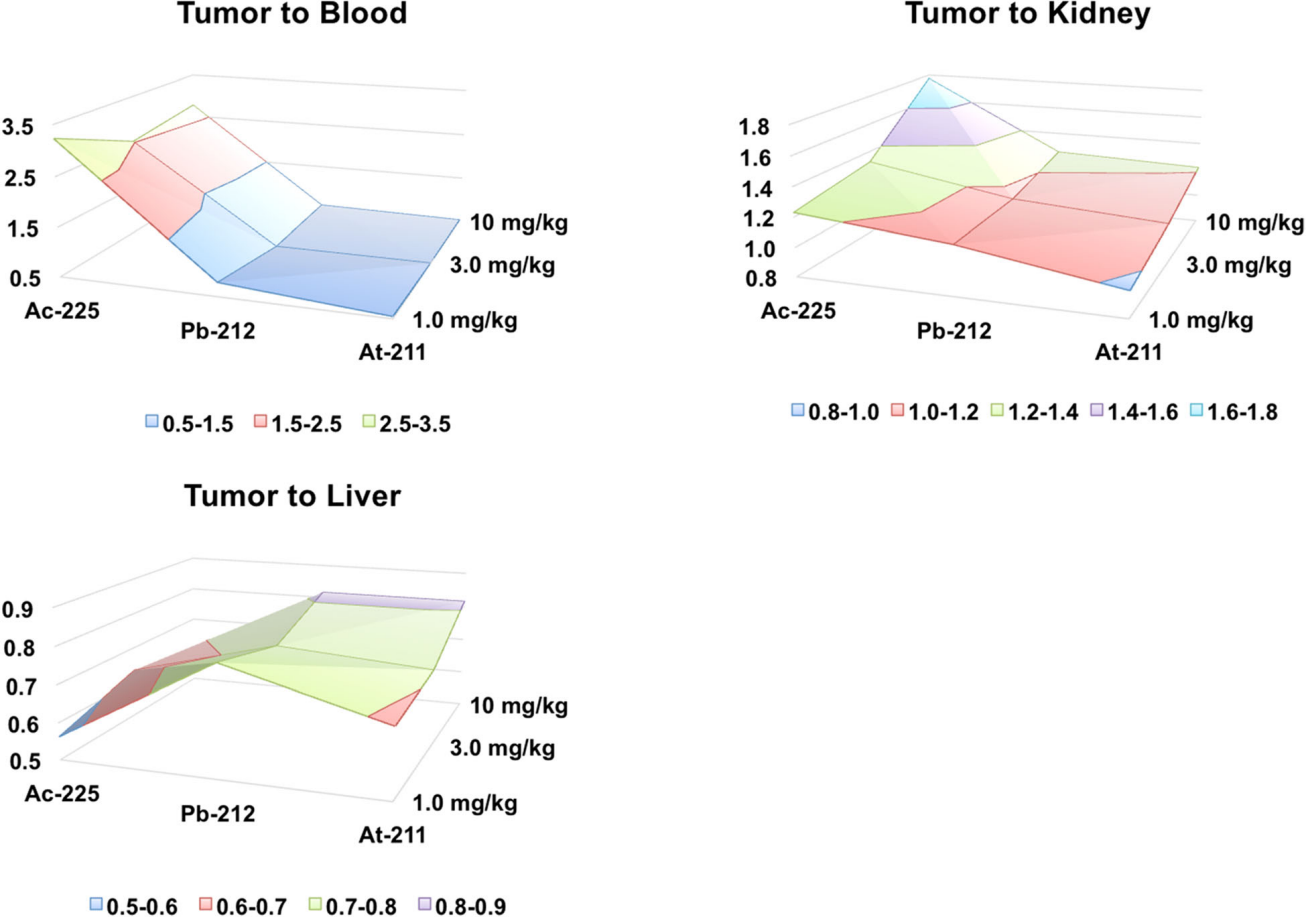
Time-integrated activity ratios (dimensionless) **a** tumor to blood (TIA-tumor/TIA-blood), **b** tumor to kidney (TIA-tumor/TIA-kidney), and **c** tumor to liver (TIA-tumor/TIA-liver) for ^225^Ac-, ^212^Pb-, and ^211^At-labeled anti-PD-L1-BC for the 1.0, 3.0, and 10 mg/kg protein concentrations

In radiopharmaceutical therapy, it is generally considered important to match the radionuclide half-life with the clearance kinetics of the agent. We used the ^111^In-DOTA-anti-PD-L1 Ab conjugate kinetics at the different administered Ab doses to approximate the tumor to normal organ dose ratios for ^225^Ac-, ^212^Pb-, and ^211^At-labeled anti-PD-L1 Ab from organ time-integrated activity (TIA) calculations (Fig. 11). The resulting tumor to normal organ ratios confirms the importance of using a long-lived emitter for radiolabeled Ab conjugate therapy. The TIA is highest in the blood for the short-lived radionuclides ^212^Pb and ^211^At. For the long-lived radionuclide, ^225^Ac, the largest TIA was in the spleen for the 1 and 3 mg/kg and in the liver for the 10 mg/kg protein doses (Fig. 12).

#### Max tolerated dose (MTD)

**Fig. 13:**
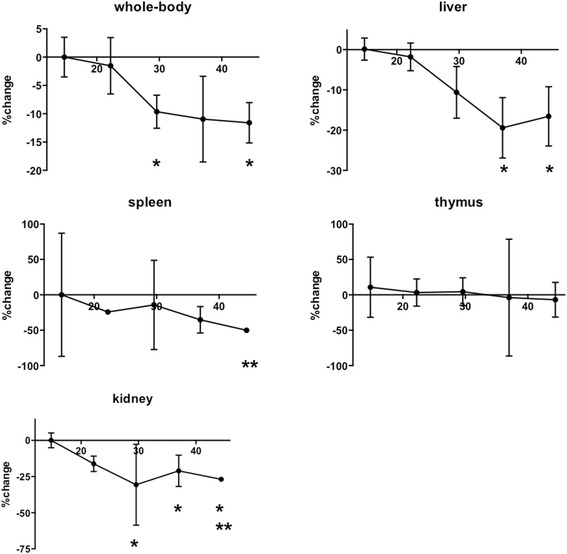
Percent change relative to the 15-kBq-treated mice in the average whole-body and organ weights of tumor-free mice treated with ^225^Ac-anti-PD-L1 (3 mg/kg, 60 μg) at increasing administered activities of 15, 22, 30, 37, and 44 kBq (*n* = 4 to 5 mice/point). *p ≤ 0.01 relative to 15 kBq; **p ≤ 0.01 relative to 22 kBq

Healthy *neu*-N mice were administered ^225^Ac-DOTA-anti-PD-L1-BC (3 mg/kg) with activities ranging between 15 and 44 kBq. All the mice were sacrificed after a 100-day monitoring period except one, which was sacrificed on day 98. Over the 100-day period, the whole-body weight of the mice decreased in a dose-dependent manner with greater weight loss at the larger administered activities (Fig. 13). Relative to the 15-kBq dose, significant decreases were seen at the 30 and 44 kBq doses (*p* ≤ 0.01). Administered activity-dependent decreases in organ weights were also observed for the spleen, kidneys, liver, and thymus. Significant decreases occurred in the spleen at the 44-kBq dose compared to the 22-kBq dose (*p* ≤ 0.001), as well as in the liver at the 37-kBq dose and 44-kBq dose as compared to the 15-kBq dose (*p* ≤ 0.01). A 20 to 40% reduction in kidney mass occurred at 22 to 30 kBq. Relative to the 15-kBq dose, significant decrease were seen at the 30 (*p* ≤ 0.007), 37 (*p* ≤ 0.005), and 44 kBq (*p* ≤ 0.0001) dose. In addition, a significant decrease (*p* ≤ 0.006) was seen at 44 kBq relative to the 22-kBq dose. Mice reaching the 100-day endpoint had blood chemistries within the normal range (Table 3) with the exception of an elevated ALP for both the 15- and 44-kBq dose. Pathology findings on *neu*-N mice receiving 15 kBq suggest mild liver, kidney, and hematopoietic/immunopoietic effects, while four out of five mice receiving 44 kBq displayed mild to modest hematopoietic/immunopoietic effects.

**Table 3:** Blood chemistries of non-tumor-bearing *neu*-N mice treated with 15 and 44 kBq of ^225^Ac-DOTA-anti-PD-L1 (3 mg/kg, 60 μg)

	Normal range^a^	15 kBq	44 kBq	44 kBq^b^
BUN (mg/dL)	8–33	19.7 ± 4.04	29.3 ± 5.74	20.0
ALP (IU/L)	35–96	122 ± 20.6	151 ± 38.6	526
T-Pro (g/dL)	3.5–7.2	6.30 ± 0.35	6.18 ± 0.36	7.2
ALT (IU/L)	17–77	60.0 ± 26.5	49.5 ± 56.4	>1000
Cre (mg/dL)	0.2–0.9	0.63 ± 0.15	0.58 ± 0.13	0.60
Calcium (mg/dL)	7.1–10.1	8.00 ± 0.30	8.56 ± 0.56	8.90
*N*	N/A	3	4	1

^a^Reference values from www.ahc.umn.edu/rar/refvalues.html

^b^Mouse was sacrificed at 98 days instead of the 100-day endpoint

The single mouse (44 kBq) that was sacrificed at 98 days displayed signs of pain, particularly labored breathing and a hunched back. Upon necropsy, the mouse was found to have a visually enlarged thymus and spleen, fluid appeared to have accumulated in the chest cavity, and the liver was pitted. The blood chemistries of this mouse all fell within the normal range, except the ALP and ALT. The ALP (526 IU/L) was again elevated outside the normal range and was significantly higher compared to the other 44-kBq-dosed mice (151 ± 38.6, *n* = 4). The ALT was over 1000 IU/L with the normal range being 17–77 IU/L. Pathology showed significant degenerative and proliferative changes in the liver and kidney, as well as hematopoietic neoplasia in this mouse.

## Discussion

Previously, we have demonstrated that an Ab against PD-L1 can be modified to deliver a radionuclide, specifically ^111^In for SPECT imaging and that the distribution of this agent is heavily impacted by the spleen [20]. Using the same strategy, we modified an anti-PD-L1 Ab to deliver ^225^Ac for targeted alpha-particle therapy as well as ^111^In to use as a surrogate-imaging agent. The objective of dosimetry is to guide the design and implementation of radiopharmaceutical therapy, either on a patient population basis or, if the variability is sufficiently high, on an individual patient basis. Key to this is that absorbed dose estimates predict likely toxicity and tumor response to treatment. It is not possible to administer a tracer level of activity in alpha-particle emitter therapy to obtain the pharmacokinetics required for dosimetry. Preclinical studies of the type described in this report must be combined with surrogate imaging agents to establish safe starting activity levels for phase I dose escalation trials.

**Fig. 14:**
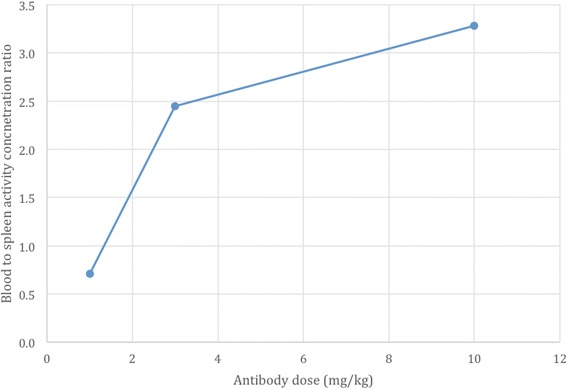
Impact of protein dose blood to spleen activity concentration ratio at 6 h post-injection of ^111^In-anti-PD-L1-BC

The surrogate-imaging agent, ^111^In-DTPA-anti-PD-L1-BC, allowed us to gauge the impact of the Ab protein concentration on the distribution of these agents, helping to select an optimal concentration to evaluate the targeted alpha-particle therapy agent, ^225^Ac-DOTA-anti-PD-L1-BC. At 6 h p.i. of 1 mg/kg Ab dose, more than 40% of the Ab per organ weight was in the spleen and 30% was in the blood giving a spleen to blood ratio of 0.7. At 3 and 10 mg/kg, the corresponding ratios are 2.4 and 3.3, suggestive of a saturation effect (Fig. 14). The high splenic uptake observed with this Ab is typically only observed with anti-leukemia Ab in the setting of a high leukemia burden in the spleen [38, 39]. The range of protein doses examined in the biodistribution studies overlaps with the range examined in human studies of anti-PD-L1 Ab, which showed the blood area under the curve (AUC) increasing linearly as a function of administered Ab [2]. The AUC reported in the clinical trials for 10 mg/kg was approximately 16-fold greater than the AUC for the 1 mg/kg dose; using the blood kinetic parameters in Table 1 to calculate AUC, the corresponding increase in our mouse model was 2.4. There was a substantial difference in clearance half-life from the circulation with the pooled mean serum half-life of the Ab, unconjugated, in the clinical studies being 15 days while the half-lives presented here of ^111^In-DTPA-anti-PD-L1 at varying doses are on the order of 20 to 50 h. This range of half-lives is consistent with clinical studies of radiolabeled Ab, while the 15-day half-life seen in the clinical study is consistent with the half-life of unconjugated antibodies [40]. Alternatively, the difference could reflect mouse to human differences in native Ab clearance kinetics.

Pre-therapy imaging for dosimetry and treatment planning is not feasible for alpha-particle emitter therapeutics due to the low administered activity, which is in contrast to beta-particle emitter therapeutics. Instead a surrogate-imaging radionuclide is required, which can result in pharmacokinetic differences. The surrogate-imaging ^111^In-DTPA-anti-PD-L1-BC demonstrated pharmacokinetic differences as compared to ^225^Ac-DOTA-anti-PD-L1-BC, particularly in the liver, kidneys, and tumor. These differences in the kidneys and liver could be attributed to the variances in the clearance and catabolism of the chelates on the Ab conjugate, ^225^Ac-DOTA chelate vs ^111^In-DTPA chelate [41]. The differences in tumor kinetics are likely impacted by the kidney and liver pharmacokinetic differences as well as differences in Ab availability arising from the lower blood time vs concentration curve.

The microscale distributions of ^225^Ac-DOTA-anti-PD-L1 Ab was obtained to better understand its therapeutic efficacy and toxicity. Uniform distribution was seen in the liver, but a non-uniform distribution was found in the spleen, thymus, and tumor. In addition, uniform distribution was also seen in the kidney of ^225^Ac-DOTA-anti-PD-L1 Ab, but previously, we have shown that ^213^Bi, a daughter of ^225^Ac, accumulates in the proximal tubules, giving a more non-uniform dose distribution overall [32, 42]. The high spleen uptake of anti-PD-L1 Ab appears to be associated with the white pulp, a PD-L1-expressing lymphocyte-rich region of the spleen. In addition, thymic uptake is also consistent with localization to lymphocyte-rich regions, predominately the PD-L1 rich cortex. Due to the heterogeneous distribution, the development of microscale dosimetry models for these organs would provide absorbed dose estimates that are more likely to relate to biologic response. It should be noted that thymic dose is less of a concern since this organ becomes largely non-functional during and after adolescence. The highly non-uniform distribution of ^225^Ac-DOTA-anti-PD-L1-BC in tumor is consistent with poor Ab penetration of all tumors.

The macroscale dosimetry calculations suggest a tolerable administered activity of 38 kBq, which would yield a 5.3-Gy tumor absorbed dose. The organ and tumor absorbed doses were calculated without accounting for the microscale dosimetry. Given the relatively uniform microscale distribution of activity within the liver at each time point, we expect from macroscale dosimetry calculations that the liver will be the dose-limiting organ. At 37 kBq and above (i.e., at the liver toxicity threshold of 28 to 32 Gy), significant liver toxicity was noted, including a significant reduction in liver mass and weight loss. In this case, the macroscopic absorbed dose calculation is consistent with the observed MTD. However, the activity distribution in the spleen and thymus was highly non-uniform; correspondingly, only the highest administered activity led to a significant reduction in spleen weight, and the thymus seemed to be insensitive to administered activity. The threshold for renal toxicity occurs at 18 to 23 Gy which corresponds to 130 to 170 kBq administered activity. As is evident from EMS. 8, renal toxicity as indicated by mass reduction occurs at far lower administered activities. This again highlights the importance of dosimetry calculations that account for the microscopic localization of alpha-particle emitters. In the case of kidneys, 63% of the absorbed dose is due to free ^213^Bi, which is predominantly localized in the renal cortex [42]. It should be noted that the absorbed dose values reported herein were not multiplied by a relative biological effectiveness (RBE) factor. A Department of Energy workshop on alpha-particle emitter therapy recommended an RBE of 5 pending the collection of additional data to identify the most appropriate RBE [43]. Preclinical measurements of RBE range from 1 to 14 [44]. Given the wide variability and lack of consistency in RBE values, we have chosen to report all doses as absorbed dose with the unit gray.

## Conclusions

Targeted alpha-particle emitting radionuclides are extremely potent over a short range, making it a valuable treatment for treating single cells and small metastatic cell clusters. The development of PD-L1-targeted high LET agents has the potential to eradicate small metastatic sites and theoretically has an immunostimulatory effect in larger PD-L1-positive tumor sites. However, these agents also target PD-L1-positive immune cells potentially limiting their use as a therapeutic agent. Both imaging and therapy using radiolabeled antibody-based checkpoint inhibition therapy will be influenced by binding to PD-L1-positive sites in normal tissues. Such normal organ uptake may be overcome to some extent by optimizing the protein amount of antibody administered for therapy, helping to protect normal tissues and potentially preserve immune cells by saturating non-tumor PD-L1 sites. An accounting of the microscale distribution of the antibody in preclinical studies is essential to the proper interpretation of organ absorbed doses and their likely relation to biologic effect. The agent developed here, ^225^Ac-DOTA-anti-PD-L1-BC, successfully targeted a PD-L1-positive tumor for delivery of ^225^Ac. However, the low absorbed dose and highly non-uniform distribution in the tumor supports the use of this agent to supplement anti-PD-L1 therapy through the recruitment of immune cells to the tumor microenvironment.

The distinction between an imaging agent used to evaluate target expression for patient selection and therapeutic response monitoring and a surrogate agent used for treatment planning is important. For the latter, the surrogate-imaging agent that best matches the pharmacokinetics of the therapeutic agent is required. Alternatively, differences in pharmacokinetics must be modeled to derive the kinetics of the therapeutic agent. In the former, a low-molecular-weight imaging agent that rapidly images PD-L1 expression in tumor sites with quick clearance is appropriate. Such an agent was recently reported and its biodistribution investigated in immunodeficient NOD-SCID (NSG) mice with xenografted tumors [45].

## Abbreviations

Ab: Antibody
BAT: Brown adipose tissue
BSA: Bovine serum albumin
FBS: Fetal bovine serum
H&E: Hematoxylin and eosin
MTD: Max tolerated dose
OCT: Optimal cutting temperature
PAS: Periodic acid-Schift stain
PD-L1: Programmed death ligand 1
PD-L2: Programmed death ligand 2
TAM: Tumor-associated macrophage
